# Development and Effect of Child Obesity Management Program by Applied Nudge

**DOI:** 10.3390/ijerph191912692

**Published:** 2022-10-04

**Authors:** Yoonji Park, Jihyun Kim

**Affiliations:** 1 Department of Nursing, Shinsung University, Dangjin-si 31801, Korea; 2 Department of Nursing, Daejeon University, Daejeon 34520, Korea

**Keywords:** child, obesity, body mass index

## Abstract

Background: Child obesity rates are increasing worldwide. In Korea, the proportion of overweight students has steadily increased from 21.8% in 2015 to 25.8% in 2019. Childhood obesity causes mental problems, such as depression and social phobia, due to mental stress, feelings of inferiority, and low self-esteem. Methods: This experimental study aimed to verify the effect of the child obesity management program on body changes (height, weight, obesity degree, body mass index [BMI], body fat percentage), eating habits, exercise habits, obesity knowledge, and social support. This child obesity management program applies the nudge technique based on an ecological model and induces autonomous weight management through environmental control. Results: As results of this study, the child obesity management program using the nudge technique developed in this study improved the height (t = −5.19, *p* < 0.001), obesity degree (z = −3.28, *p* = 0.001), BMI (z = −3.22, *p* = 0.001), exercise habits (t = −2.09, *p* = 0.040), and obesity knowledge of obese children (z = −2.99, *p* = 0.003). Conclusions: This multidimensional intervention improved obesity by inducing and sustaining behavioral changes in obese children. Therefore, applying the nudge techniques and multidimensional intervention methods based on ecological model are proposed to increase the effectiveness of the health promotion programs.

## 1. Introduction

Child obesity rates are increasing worldwide. Over the past 40 years, from 1975 to 2016, the number of obese children and adolescents has increased more than 10 times, from 11 million to 120 million [1]. Accordingly, the World Health Organization (WHO) regards childhood obesity as a serious health problem and insists that it be actively dealt with [2].

In Korea, due to health examinations from elementary to high school, the proportion of overweight students has steadily increased from 21.8% in 2015 to 25.8% in 2019 [3] Childhood obesity causes mental problems, such as depression and social phobia, due to mental stress, feelings of inferiority, and low self-esteem [4]. These negatively affect physical, psychological, emotional, and social growth and development, causing adult obesity and increasing the risk of various diseases [5].

Various factors are related to obesity in children including socioeconomic factors, lifestyle, and diet [6], and parental lifestyle [7]. School age is a period in which overall lifestyle habits are established [8], and it is essential to acquire the right health habits during this period. Schools are important environments where children spend a lot of time with meaningful people, such as friends and teachers. Thus, health promotion programs in schools are important for the education and health promotion of students: they help school-aged children from healthy habits [9].

To solve the problem of obesity in children, it is necessary to understand their characteristics and find solutions suitable for those characteristics. It has been reported that the degree of children‘s perception can change even with small environmental changes, and there is not much accumulation of empirical knowledge compared to adults; therefore the influence of visual factors in recognizing environmental information is greater [10,11]. Environmental changes through visual stimulation can effectively prevent and manage obesity in children.

The nudge technique, which has recently been used in various fields, has been evaluated as an efficient and non-compulsive method for inducing an individual‘s healthy choice among visual stimuli. One example of the nudge technique is that by attaching a fly sticker to the toilet in the men‘s bathroom, the amount of urine sticking out around the area was reduced by approximately 80%, thereby improving hygiene [12].

The ecological model assumes that individual behavior changes through interaction with the environment. In this theory, the environment surrounding individuals is systematically classified into individual, organizational, community, and public policy factors, and presents a direction for establishing an integrated arbitration strategy [13]. Children‘s obesity problems should be based on environmental changes, such as parents, teachers, friends, and school environments surrounding the individual and the individual. An obesity management program based on an ecological model can be an effective intervention program to approach and manage factors affecting obesity systematically.

Therefore, this study attempted to develop a child obesity management program by applying a nudge technique based on an ecological model and verifying its effectiveness [13].

## 2. Materials and Methods

### 2.1. Methods

This experimental study aimed to verify the effect of the child obesity management program on body changes (height, weight, obesity degree, body mass index (BMI), body fat percentage), eating habits, exercise habits, obesity knowledge, and social support. The study used a non-equivalent control group pretest-posttest design.

### 2.2. Study Participants

This study was conducted with two elementary school (Grade 5 students) in a city in South Korea. The two selected elementary schools were located more than 20 km apart, had similar environmental and economic conditions, and did not have their own obesity-related intervention programs. The two schools selected have statistically similar population numbers and economic status.

**Figure 1:**
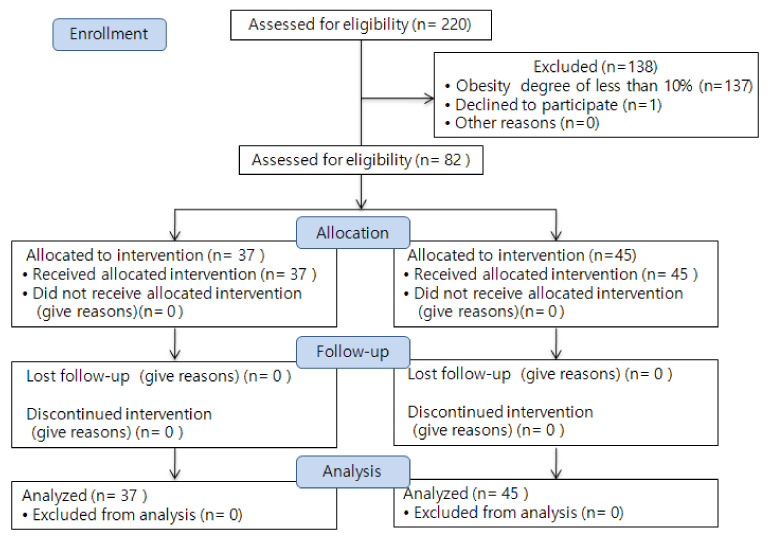
Flow of participants through the trial.

Each school was assigned to an experimental group or a control group. Based on previous studies [14], the number of study participants was set at a significance level of α = 0.05, power 1-β = 0.8, and effect size d = 0.80. In the G-power 3.1.5 program, the number of participants required for each group was 21. To control for exogenous variables, the program was applied to all students in one grade (experimental group: 98 and control group: 122), and consent for program participation was obtained from both students and parents. Among all students who agreed to participate in the program, only data from students with obesity degree of 10% or higher (experimental group: 37 and control group: 45) were used to analyze the results (Figure 1).

### 2.3. Measures

#### 2.3.1. Obesity Degree

Based on the Korean Pediatric Body Development Standard [15] of The Korean Pediatric Society, the 50th percentile of weight by height was set as the standard weight, and the obesity degree was calculated (Obesity Degree% = (actual weight—standard weight for each height)/standard weight for each height × 100). Depending on the value, from −10% to less than 10% are defined as normal, from 10% to less than 20% are overweight, and 20% or more are defined as obese. Obesity shows the degree of obesity that reflects the characteristics of Korean children rather than BMI [15].

#### 2.3.2. BMI (Body Mass Index)

BMI was calculated by dividing weight by the square of height as shown in the following formula. (BMI) = Weight (kg)/Height (m^2^).

#### 2.3.3. Body Fat Percentage %

Body fat percentage was measured with a body composition analyzer (Inbody J05; Biospace, Seoul, Korea) using bioelectrical impedance.

#### 2.3.4. Eating Habits

This refers to the score measured by the ‘Eating Habit Survey Tool’ in the study of Sora Park [16]. The eating habit tool consists of 30 questions that include questions such as “I eat something when I watch a book or TV” and “If there is something delicious after a meal, I eat again.” Each item was measured using a Likert five points scale, with scores ranging from 30 to 150 points. A higher score indicated better eating habits. In Sora Park‘s study [16], Cronbach‘s α was 0.86, and Cronbach‘s α in this study was 0.93.

#### 2.3.5. Exercise Habits

We used the ‘Exercise Habit Survey Tool’ of Jeon Misook [17], modified and improved by Sora Park [16]. This tool consists of 12 questions related to usual exercise habits, such as “I have regular exercise” and “I tend to use stairs rather than elevators.” Each item was measured using a Likert five points scale, with scores ranging from 12 to 60 points. A higher score indicated better exercise habits. In Sora Park‘s study [16], Cronbach‘s α was 0.86, and Cronbach‘s α in this study was 0.78.

#### 2.3.6. Obesity Knowledge

In this study, we used the ‘Obesity knowledge tool’ of Jeon Misook [17], modified and improved by Sora Park [17]. The obesity knowledge tool consisted of questions O and X, which marked O what they thought was correct and checked whether the child’s knowledge related to obesity was correct. A higher score indicated better knowledge of obesity. At the time of tool development, Cronbach‘s α was 0.78. In this study, it was 0.70.

#### 2.3.7. Social Support

The Multidimensional Scale of Perceived Social Support (MSPSS) tool developed by Zimet et al. was used [18], with a total of 12 items (four questions each asking for family support, support from friends, and the support of major others). Each item was measured using a Likert five points scale, with higher scores indicating better social support. In this study, Cronbach‘s α for family support was 0.84, Cronbach‘s α for friend support was 0.86, and Cronbach‘s α for major others was 0.88.

### 2.4. Research Process

#### Development and Application of the Child Obesity Management Program

This child obesity management program applies the Nudge technique based on an ecological model and induces autonomous weight management through environmental control.

According to Sora Park [16], obesity-related factors were classified according to the dimensions of the ecological model, and programs were organized accordingly. As individual factors, eating habits, exercise habits [16], and obesity knowledge [19] were selected as intervention factors. As interpersonal factors, social support from friends, parents, and teachers was selected [20,21,22]. Organizational factors included the amount of school activity, the characteristics of school meals, and school policy changes [19,23]. An obesity management program linked to a public health center was selected as the community factor.

Interventions for changing individual factors included setting healthy weight through a pledge to maintain health, education related to eating and exercise habits, and self-assessment sticker activities for healthy eating and exercise (Table 1). Education related to eating habits and exercise was conducted as group education for each class and was conducted three times for 40 min each. To motivate people to practice healthy eating and exercise and to enhance self-efficacy, they were instructed to attach a sticker after practicing a healthy diet and exercise, and then rewarded. In order to prevent the decrease in self-esteem of obesity children who participated in the study, an intrapersonal program and parent education were implemented, including both obese children and non-obese children. Therefore, the program and education were applied to all students in one grade. When analyzing the data, only the data of obesity children were analyzed.

Interpersonal interventions included parental education, teacher education, and the improvement of social support (Table 1). Parent education was conducted five times, and exercise and diet education materials were distributed through parent letters, and a traffic light diet table was provided to encourage healthy eating practices. The teachers were educated on child obesity prevention and program progress. We also attempted to increase the social support of obese children by ensuring all students and teachers in the same grade participated in the exercise.

Organizational factor interventions included environmental changes (health murals) in schools, which are the main living spaces of students, and organized exercise and diet control programs as the school’s regular programs [24]. Based on meta-analysis [25], the exercise program was organized as follows: 30 min of aerobic exercise for at least 9 weeks, 5 times a week, and regular exercise during lunchtime [25]. In order to induce students to eat the right amount of food, the entire school meal tray was replaced with a rainbow meal tray. The rainbow meal tray has a line on its bottom to indicate the amount of food, and the line is drawn like a rainbow. It helps each person know the right amount of their food and eat it without overeating. The rainbow meal tray used in this program was a meal tray indicating the appropriate amount of food for elementary school students. The order of placing food was changed so that vegetables could be placed first. To motivate healthy eating and exercise, health murals were installed using the entrance, stairs, and corridors walls of the school (Table 1).

As an intervention for community factors, obesity management programs, including professionals (exercise specialists and, nutritionists) at local public health centers, were developed and distributed (Table 1). In addition, the Health Care Golden Bell Program (quiz competition) was implemented as a public health center-linked program. At the local health center, an exercise specialist, a nutritionist, and daily program managers were dispatched to the school to run the program in this program.

Nudge techniques were applied to the developed child obesity management program. The nudge technique induces behavior by self-determination rather than external forces or coercion. Thus, behavior can be maintained for a long time [24]. The nudge technique applied in this study included installing health murals in corridors and stair walls, using rainbow meal tray to indicate the quantity of meals [26], and changing the order of food to place fruits and vegetables first (nudge reordering technique) during meals (Table 1). In addition, the opt-out system, one of nudge’s policy design methods, was used to set regular exercise times and exclude only students who expressed their intention not to attend [27].

Diet and exercise education, activities with self-evaluation stickers, parent and teacher education, and exercise programs were applied for 9 weeks. In addition, rainbow plates, changing the order of food during meals, and health murals continued even after the program was over.

The child obesity management program was verified by six professionals, including obesity exercise specialists, for the validity of the educational content and intervention method, and the content validity index (CVI) was 0.89.

## 3. Results

### 3.1. The General Characteristics and the Homogeneity of the Research Variables

There were 37 participants in the experimental group, 24 male students (64.9%), 13 female students (35.1%), and 45 in the control group, 27 male students (60.0%), and 18 female students (40.0%). The participants who had tried to lose weight were 31 (83.8%) in the experimental group and 36 (77.8%) in the control group. As a result of verifying the homogeneity of the general characteristics of the participants, there was no significant difference between the two groups, confirming that the two groups were homogeneous (Table 2).

### 3.2. Body Changes Related to Obesity

After implementing the program, the height increased by 1.95 cm in the experimental group and 1.10 cm in the control group, and the difference between the two groups was statistically significant (t = −5.19, *p* < 0.001). Obesity degree decreased by 3.62% in the experimental group and 0.58% in the control group, showing a statistically significant difference (z = −3.28, *p* = 0.001). BMI before and after the intervention was changed by 0.42 in the experimental group and 0.05 in the control group, showing a statistically significant difference (z = −3.22, *p* = 0.001). Body fat percentage and body weight were not statistically significantly different (Table 3).

### 3.3. Eating Habits and Exercise Habits

The eating habit scores before and after the intervention did not show a statistically significant difference (z = −0.84, *p* = 0.401). Exercise habits before and after the intervention changed by 2.00 points in the experimental group and −0.05 points in the control group, showing a statistically significant difference (t = −2.09, *p* = 0.040) (Table 4).

### 3.4. Obesity Knowledge and Social Support

Obesity knowledge before and after the intervention increased by 1.76 points in the experimental group and 0.62 points in the control group, and the results showed a statistically significant difference (z = −2.99, *p* = 0.003). Social support pre-intervention and post-intervention increased by 2.70 points in the experimental group and 1.82 points in the control group, but it was not statistically significant (z = −0.15, *p* = 0.881) (Table 5).

## 4. Discussion

The child obesity management program using the nudge technique developed in this study not only improved the height, obesity degree, and BMI of obese children, but also improved exercise habits and obesity knowledge.

After implementing the child obesity management program, the obesity-related indices and BMI of the target children significantly changed. After the intervention, obesity decreased by 3.62% in the experimental group and 0.58% in the control group, and BMI showed changes in the experimental group −0.42 and the control group 0.05. These results confirm that the obesity management program using the nudge technique effectively reduces obesity degree in obese children. These results are similar to those of a previous study [1] that applied an eight-week obesity management program to obese children. The obesity management program applied in this study was constructed based on an ecological model. Education on eating habits and exercise habits was provided to change individual factors, and teachers and parents were educated to change interpersonal factors. The nine-week exercise program was applied to change the organizational factors, and health murals were installed an entrance, stairs, and corridors walls of the school. To induce changes in eating habits, the order of food placement in the cafeteria was changed, and the meal tray was changed to a rainbow meal tray. As part of the health promotion program of the local public health center, child obesity management program was disseminated, and educational materials were provided to change community factors. This multidimensional intervention improved obesity and BMI by inducing and sustaining behavioral changes in obese children. In addition, it is believed that the intervention method applying nudge techniques, such as the change in the order of placing food in the cafeteria and the use of rainbow meal tray, induced and maintained the change in behavior by self-determination. In previous studies [28], applying the nudge technique to a health promotion program, it was also reported that the participants‘ health habits changed. This nudge technique can promote behavior change and maintenance based on freedom of choice, rather than the use of force [29]. Therefore, applying the nudge technique is proposed to increase the effectiveness of health promotion programs. In this study, it is analyzed that the growth of the participant’s height affected the decrease to BMI. Previous studies conducted weight management program including exercise program for obesity children also showed a significant increased height along with a decreased BMI [30]. This growth of height is analyzed as the effect of the exercise program included in the obesity management program, and it is estimated that periodic exercise conducted for nine weeks promotes children’s growth hormones and induces height growth.

However, in this study, there was no significant change in the body fat rate of obese children after implementing the program. This result is contrary to previous studies [29,30] that applied the obesity management program for 10–12 weeks, which was found to be related to the application period of the obesity management program. The American College of Sports Medicine (ACSM) [5] presented at least 8–12 weeks of exercise to reduce body fat. Furthermore, a meta-analysis study [25] that analyzed the exercise program explained that the longer the program application period, the more effective the change in body fat. Unlike obesity degree and BMI, which are measured based on weight and height, changes in the body fat rate in the actual body composition ratio. Therefore, exercise and management programs should be applied steadily for more than 10 weeks. We recommend a study on obesity programs using the nudge technique for more than 10 weeks to confirm changes in body fat rate, obesity degree, and BMI in obese children.

In this study, the height of the participants increased significantly after nine weeks of program application. These results were analyzed as an effect of the exercise program included in the child obesity management program. The exercise program applied in this study was conducted five times a week for 30 min for nine weeks, and walking at a constant speed was conducted in the playground. Periodic exercise has been reported to promote growth hormone levels [31]. During exercise, the secretion of growth hormone is increased up to 25 times compared to that during rest [32], and the secretion of growth hormone increases with the start of exercise and is highest 30 min after exercise and remains high until 90 min after exercise [31]. In addition, the exercise program conducted in this study involved walking on playground exposed to sunlight, which is a factor that induces vitamin D synthesis, stimulates the secretion of growth hormone, and stimulates calcium and bone metabolism.

After the program, the participants exercise habit scores increased significantly. These results are consistent with the results of 12 weeks of obesity management programs applied to elementary school children and improved exercise habits. This change in exercise habits can be analyzed as an effect of the exercise program applied for nine weeks. A previous study analyzing the effect of an exercise program [33] suggested that changes in eating and exercise habits may vary depending on the duration of the exercise program. In this study, the participants exercise habits improved significantly, but their eating habits did not. Unlike exercise habits, eating habits have characteristics formed in infancy or childhood and fixed in adolescence [34]. The influence of parents who are food providers is so great that it takes a lot of time to change them, and it is difficult to change them only with child intervention [10]. In this study, parental education was conducted to change children‘s eating and exercise habits; however, after the intervention, exercise habits improved, but eating habits did not. These results suggest the need for a study to develop a nine-week or longer obesity management program that strengthens parental education and verifies effectiveness.

This study has a limitation in that the research results cannot be generalized to all children because the study subjects were only students from two schools in some regions. In addition, in order to prevent the decrease in self-esteem of obesity children who participated in the study, the intervention program was implemented, including both obesity children and non-obesity children. However, there is a possibility that the participants were exposed during the data collection process. Accordingly, a study is suggested to identify emotional problems, such as self-esteem reduction and stigma of children who participated in the obesity management program. This study compared the differences before and after program intervention between the experimental group and the control group to verify the effectiveness of the child obesity management program. However, this study has a limitation in that it cannot compare the differences according to the demographic characteristics of children and parents. Accordingly, a follow -up study was proposed to verify the effectiveness of the child obesity management program according to the demographic characteristics of children and parents.

## 5. Conclusions

This study attempted to develop a child obesity management program and verifying its effectiveness. This child obesity management program applies the nudge technique based on an ecological model and induces autonomous weight management through environmental control. As results of the study, the child obesity management program using the nudge technique developed in this study not only improved the height, obesity, and BMI of obese children, but also improved exercise habits and obesity knowledge. This multidimensional intervention improved obesity by inducing and sustaining behavioral changes in obese children. Therefore, applying the nudge techniques and multidimensional intervention methods based on ecological models is proposed to increase the effectiveness of the health promotion programs.

## Figures and Tables

**Table 1:** Child Obesity Management Program with Nudge Based on Ecological Model.

Sources	Title	Contents	Time
Intrapersonal	Healthy weight setting	Setting healthy weight using self-declaration	1 time, 40 min. At the start of the program
	Diet education	Healthy Eating and Traffic Light Diet Education	1 time, 40 min.
	Exercise education	Weight training, aerobic exercise	1 time, 40 min.
	Self-evaluation sticker activity	Self-assessment sticker activity after exercise and diet	Once a day, During the program period
interpersonal	Parent education (Parents letters)	Child obesity prevention, diet and exercise education	5 times
	Teacher education	Child obesity prevention, Explanation of this program’s progress	1 time, 40 min.
	Improving social support	All students of that grade participate in the program	During the program period
Organization	Health Stairs mural installation	School entrance, the walls of stairs and corridors	Throughout school life
	Exercise program	Walking on the playground after lunch	9 weeks, 5 times a week, 30 min at a time
	Provision of rainbow plates	A plate to check the proper amount of food	Every lunch at school
	Change the location of food	Reordering food positions in the cafeteria to put fruits and vegetables first	Every lunch at school
Community	Development and Dissemination of Obesity Management Programs linked to Local Public health center	Provide public health center professionals (exercise specialist, nutritionists)	During the program period

**Table 2:** Homogeneity test and General Characteristics of the Participants (N = 82).

Characteristics	Categories	Exp. (n = 37)	Cont. (n = 45)	χ^2^ or t	*p*
		n (%) or M ± SD	n (%) or M ± SD		
Age	year	10.77 ± 0.30	10.74 ± 0.27	−0.43	0.667
Gender	Male	24 (64.9)	27 (60.0)	0.20	0.651
	Female	13 (35.1)	18 (40.0)		
Mother’s occupation status	have	29 (78.4)	28 (62.2)	2.50	0.114
	none	8 (21.6)	17 (37.8)		
Self-body image	moderation	9 (24.3)	11 (24.4)	1.15	0.561
	A bit obese	17 (46.0)	25 (55.6)		
	A lot of obesity	11 (29.7)	9 (20.0)		
Obese people in the family	have	17 (45.9)	23 (51.1)	0.21	0.641
	none	20 (54.1)	22 (48.9)		
Attempts to lose weight	have	31 (83.8)	35 (77.8)	0.50	0.582
	none	6 (16.2)	10 (22.2)		
Obesity management education experience	have	23 (62.2)	33 (73.3)	1.17	0.279
	none	14 (37.8)	12 (26.7)		
Walking time to and from school	minute	15.24 ± 11.38	11.89 ± 10.07	−1.42	0.161

**Table 3:** Difference in Body Change Related to Obesity (N = 82).

Variables		Exp. (n = 37)	Cont. (n = 45)	t or z(*p*)
		M ± SD	M ± SD	
Height (cm)	Pretest	146.72 ± 6.91	146.43 ± 6.95	
	Posttest	148.67 ± 6.84	147.53 ± 7.13	
	Difference	1.95 ± 0.77	1.10 ± 0.72	−5.19(<0.001)
Weight (kg)	Pretest	52.52 ± 11.54	51.90 ± 10.18	
	Posttest	52.96 ± 11.52	52.82 ± 10.80	
	Difference	0.44 ± 1.31	0.92 ± 1.49	−1.54 *(0.124)
Obesity degree (%)	Pretest	27.43 ± 14.03	26.98 ± 12.77	
	Posttest	23.81 ± 15.04	26.40 ± 14.18	
	Difference	−3.62 ± 3.76	−0.58 ± 3.91	−3.28 *(0.001)
BMI	Pretest	24.13 ± 3.28	23.99 ± 2.95	
	Posttest	23.71 ± 3.28	24.04 ± 3.19	
	Difference	−0.42 ± 0.56	0.05 ± 0.69	−3.22 *(0.001)
Body fat percentage	Pretest	36.79 ± 5.59	37.42 ± 5.76	
	Posttest	35.94 ± 5.84	36.60 ± 6.04	
	Difference	−0.85 ± 2.56	−0.82 ± 1.90	0.08(0.940)

Exp. = Experimental group; Cont. = Control group * Mann-Whitney U Test.

**Table 4:** Difference in Eating Habits and Exercise Habits (N = 82).

Variables		Exp. (n = 37)	Cont. (n = 45)	t or z(*p*)
		M ± SD	M ± SD	
Eating habits	Pretest	103.51 ± 17.10	107.71 ± 13.07	
	Posttest	107.70 ± 17.36	109.56 ± 12.75	
	Difference	4.19 ± 10.69	1.85 ± 10.29	−0.84 *(0.401)
Exercise habits	Pretest	44.16 ± 4.81	42.78 ± 5.89	
	Posttest	46.16 ± 5.79	42.73 ± 6.32	
	Difference	2.00 ± 4.47	−0.05 ± 4.34	−2.09(0.040)

Exp. = Experimental group; Cont. = Control group * Mann-Whitney U Test.

**Table 5:** Difference in Obesity Knowledge and Social Support (N = 82).

Variables		Exp. (n = 37)	Cont. (n = 45)	t or z(*p*)
		M ± SD	M ± SD	
Obesity knowledge	Pretest	22.70 ± 2.72	21.69 ± 2.40	
	Posttest	24.46 ± 1.98	22.31 ± 2.16	
	Difference	1.76 ± 3.04	0.62 ± 1.90	−2.99 *(0.003)
Social support	Pretest	45.19 ± 9.18	45.09 ± 8.33	
	Posttest	47.89 ± 7.46	46.91 ± 9.21	
	Difference	2.70 ± 6.55	1.82 ± 9.53	−0.15 *(0.881)

Exp. = Experimental group; Cont. = Control group * Mann-Whitney U Test.

## Data Availability

The datasets used and/or analyzed during the current study are available from the corresponding author on request.
